# Assessment of features in facial hyperpigmentation: Comparison study between VISIA and CSKIN

**DOI:** 10.1111/srt.13216

**Published:** 2022-10-29

**Authors:** Ying Zuo, Anqi Li, Hailun He, Ruoyu Wan, Yu Li, Li Li

**Affiliations:** ^1^ Department of Dermatology and Venereology West China Hospital Sichuan University Chengdu China; ^2^ Cosmetic Safety and Efficacy Evaluation Center of West China Hospital Sichuan University Chengdu China

**Keywords:** brown spots, CSKIN, hyperpigmentation, skin color, VISIA

## Abstract

**Background:**

Hyperpigmentary disorder is one of the commonest skin concerns in dermatology clinics. The availability of noninvasive instruments provided a convenient, objective, and reproducible methodology for the evaluation of pigmentation and skin color. The aim of this study is to compare CSKIN and VISIA in measuring facial hyperpigmentation, as well as to assess the correlation between the instrumental analyzing and clinical evaluation.

**Methods:**

Eighty Chinese patients were enrolled. Images were taken and analyzed by VISIA from Canfield and CSKIN from Yanyun Technology, and the facial hyperpigmentation was graded by three dermatologists.

**Results:**

Feature counts within the facial pigmented areas analyzed by VISIA showed positive correlations with brown pixels (*r* = 0.331, *p* < 0.05) and brown percent (*r* = 0.395, *p* < 0.0001) measured by CSKIN. The parameters measured by CSKIN and VISIA were significantly correlated with visual scores graded by the dermatologists, with VISIA presenting a moderate correlation (*r* = 0.509, *p* < 0.001) and CSKIN a slightly stronger correlation with the visual scores (*r* = 0.653, *p* < 0.001).

**Conclusion:**

CSKIN could serve as an alternative in the assessment and follow‐up of skin disease featuring with facial hyperpigmentation.

## 1 INTRODUCTION

Hyperpigmentary disorder is one of the commonest skin concerns in dermatology clinics, and making an objective and precise assessment for the severity of the disease and the efficacy of the treatment is very important in the successful management of hyperpigmentary disorders.^1^, ^2^, ^3^ Skin hyperpigmentation is a broad term to describe increased pigmentation in the skin, including melasma, solar lentigines, Nevus of Ota, Riehl's melanosis, and post‐inflammatory hyperpigmentation caused by dermatosis such as acne, contact dermatitis. Although hyperpigmentation usually does no harm to health, it could bring negative psychological impact and decrease life quality of the patient.^4^, ^5^

Visual severity scales are well recognized for clinical assessment by clinicians and evaluators and so on (the MASI scale for the evaluation of melasma).^6^ However, visual assessment could be affected by subjective factors and time‐consuming and, thus, are prone to significant interobserver variability.^7^ Therefore, quick, quantitative, accurate, and reproducible methods for measuring hyperpigmentation of multiple categories are required to improve the diagnosis and evaluation procedure as well as to provide with a reliable monitor of the progress after treatment. By means of a variety of noninvasive instruments, such as dermoscopy,^8^ Mexameter,^9^ DermaSpectrometer,^10^ Chroma Meter,^10^ VISIA,^11^ Antera 3D,^9^ and ImageJ with its specially designed plug‐in,^12^ skin hyperpigmentation can be obtained for the objective measurement of skin color.

CSKIN from Yanyun Technology, previously reported by Chen et al.,^13^ is a newly developed noninvasive instrument for the measurement of various facial skin features, including red, brown spots, acne, pores, porphyrins, UV spots, and wrinkles. CSKIN is of good capability of capturing high‐resolution images with refined image processing techniques. The algorithm of CSKIN has been recently updated, and the latest machine learning technique was used in identifying specific facial features. However, VISIA from Canfield Scientific, which is the most frequently used noninvasive measuring device for facial skin parameters in the department of dermatology and beauty clinics or cosmetic salons, is considered a beneficial tool for dermatology and esthetic practices.^14^ The aim of this study is to compare CSKIN and VISIA in measuring facial hyperpigmentation, as well as to assess the correlation between the instrumental analyzing and clinical evaluation.

## 2 METHODS

### 2.1 Study population

Eighty volunteers (8 male and 72 female, 34.6 ± 11.6 years old) were recruited from January 2 to May 18, 2019 in the department of dermatology, West China Hospital, Sichuan University. All the volunteers enrolled have given written informed consent.

### 2.2 Measurements

All measurements were performed under the controlled dark conditions (22 ± 2°C, 45% ± 5% relative humidity). Volunteers were asked to clean their face with clean water 20 min before the test. Pictures of the front, left, and right side of the face were taken by two instruments, respectively.

### 2.3 Grading by the dermatologist

Patients’ skin conditions were evaluated by three certified dermatologists using the simplified Physician Global Assessment^15^, ^16^ score scale (0 = almost clear, 1 = mild, 2 = moderate, 3 = severe) in terms of the degree of hyperpigmentation. Each of the three dermatologists had clinical experience in dermatology for more than 2 years. All the facial features of the patients were observed under the same condition, including the lighting, the background, the position of the patients, and the observing distance between.

### 2.4 Statistical analysis

Correlations between VISIA and CSKIN were determined by calculating the Pearson correlation coefficient. Spearman's rank correlation coefficient was used to determine the correlation between visual grading provided by the dermatologists and each parameter for the brownies of imaging analysis produced by VISIA and CSKIN separately. Consistency of the feature grading results by three dermatologists was evaluated by Kendall's coefficient of concordance. The data were performed by SPSS 22.0 software (IBM Corporation).

## 3 RESULTS

### 3.1 Instrumental differences between VISIA and CSKIN

**FIGURE 1:**
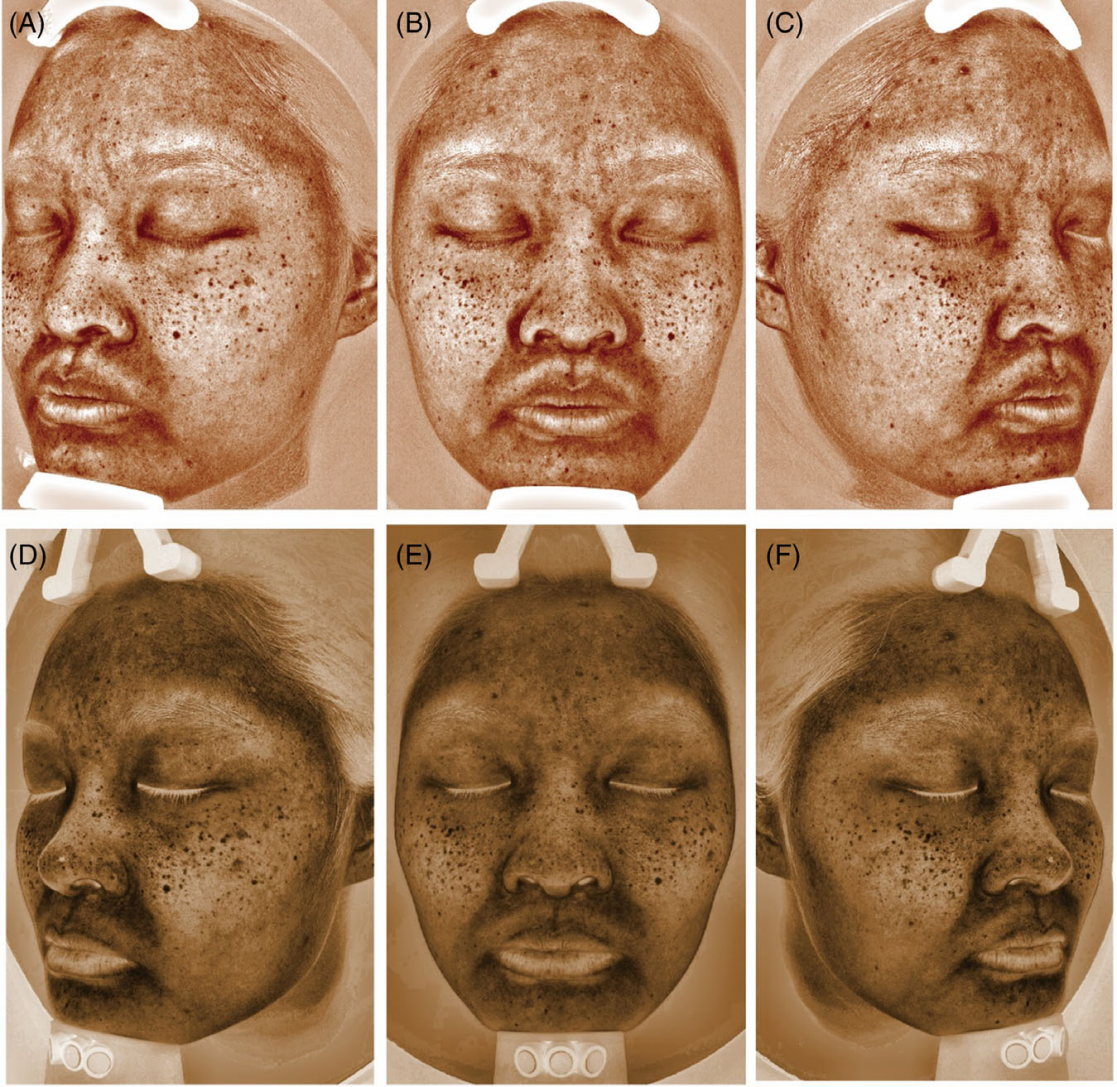
Images of pigmented area captured by VISIA (A)–(C) and by CSKIN (D)–(F)

The indices of brown spots were recorded and measured by VISIA (Figure 1A–C) and CSKIN (Figure 1D–F). A brief comparison of the two instruments was depicted in Table 1.

**TABLE 1:** Comparison of instrumental parameters of VISIA and CSKIN

Parameter	VISIA	CSKIN
Operating system	Windows	IOS
External connectivity	PC	iPad
Light sources	Standard/UV/cross‐polarized light	Standard incandescent/UV/cross‐polarized light (white/green/blue)
Color channel	RGB	LAB
Resolution (pixels)	15 million	54 million
Data deposition	Local disk	Cloud
Disk space	Minimum: 60–80 GB	None
Analyzed indices	Spots, wrinkles, textures, pores, UV spots, brown spots, red areas, porphyrins	Acne, red, brown spots, pores, porphyrins, UV spots, wrinkles
Values	Feature counts, absolute scores, percentiles	Pixels, percent (area), dots
Skin hyperpigmentation–related parameters	Brown spots feature counts, brown spots absolute scores	Brown pixels, brown percent (area)
Numbers of images	24	16

Abbreviation: RGB, red–green–blue.

### 3.2 Correlations of the facial features of hyperpigmentation between VISIA and CSKIN

**FIGURE 2:**
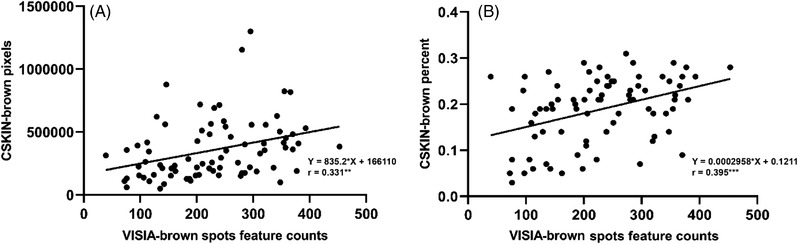
Scatter diagrams of the values within the pigmented area measured by VISIA and CSKIN

Pigmentation‐related parameters measured by CSKIN (brown pixels and brown percent) showed statistically significant correlation with VISIA (brown spots feature counts) (Figure 2).

### 3.3 Evaluated scores of skin hyperpigmentation by the dermatologists and its internal consistency

Precisely, 240 times of visual evaluation have been made in terms of hyperpigmentation by three dermatologists who were double‐blinded. Significant consistency was observed among the results evaluated by the dermatologists (Table 2).

**TABLE 2:** Facial hyperpigmentation evaluated by three dermatologists

Dermatologist	Score (mean ± SD)	Kendall *w*	*p*
A	1.09 ± 0.81	0.745	<0.0001
B	1.03 ± 0.78		
C	1.39 ± 0.72		

Abbreviation: SD, standard deviation.

### 3.4 Correlations between parameters analyzed by instruments and the visual scores graded by dermatologists

Brown spots feature counts measured by VISIA showed moderate correlation with mean visual scores graded by the three dermatologists. In contrast, brown pixels measured by CSKIN presented better consistency with the clinical grading. Brown spots absolute scores, another parameter of VISIA, revealed weak correlation with manual grading. However, no significant correlation was found between brown percent by CSKIN and visual grading (Table 3).

**TABLE 3:** Correlations of visual scores graded by dermatologists and parameters measured by VISIA and CSKIN

Parameter	*r*	*p*
VISIA‐brown spots feature counts	0.509**	0.001
VISIA‐brown spots absolute scores	0.369**	0.001
CSKIN‐brown pixels	0.653**	0.001
CSKIN‐brown percent	0.146	0.197

**p<0.01.

## 4 DISCUSSION

“Brown spots” refers to lesions on and deeper within the skin, such as post‐inflammatory hyperpigmentation, freckles, lentigines, and melasma. Skin color is predominantly determined by pigments, such as melanin, hemoglobin, bilirubin, and carotene. Skin hyperpigmentation occurs when there is a change in melanin production and/or its distribution.^17^ Melanin has a broad absorption across visible wavelengths (400–700 nm) and ultraviolet (100–400 nm).^18^ The VISIA system generates a series of photographs using standard, ultraviolet, and cross‐polarized lighting. By means of polarized light photography, the visibilities of pigmented lesions and telangiectasia are enhanced by filtering the irrelevant surface details.^8^ Therefore, theoretically, such photography system could provide us with a better view for the detection of skin hyperpigmentation. Compared with the standard flash lighting source of VISIA, CSKIN includes three LED lighting sources (white, green, and blue) that form into cross‐polarization by the imaging system. The LED lightings surpass the normal flash lighting in the presentation of brightness difference of images, with less light decay and a long service life that lasts up to 100 000 h.

Distinct operating mechanism might account for the weak correlation of the pigmentation‐related parameter between VISIA and CSKIN. VISIA is an RGB (red–green–blue)‐based device, with which the image is processed. However, CSKIN is based on the standard Commission Internationale de l'Eclairage (CIELab) color space, which is represented by three axes: L*, a*, and b*.^19^ Color detection accuracy in CIELab is dependent on three factors: (1) the illuminating light specifications, (2) the light modulation by the tissue under test, and (3) the human vision attributes.^20^

Compared with VISIA, CSKIN showed the stronger capacity of hyperpigmentation assessment according to the higher correlation with visual scores of severity graded by three clinicians. To be exact, the parameter of brown spot feature counts measured by VISIA and brown pixels by CSKIN are suggested to be the dominant reference during hyperpigmentation analysis. However, the brown spots absolute scores by VISIA are of less efficiency for evaluation. The brown percent measured by CSKIN is considered insufficient to be an effective indicator for the severity of hyperpigmentation in reference to the inconsistency with visual scores. We assume that different color spaces could partly explain the varied correlation with visual grading between the two instruments. Due to the optical properties of skin that is spectrally dependent on the encompassed chromophores, including both types of hemoglobin and melanin, it is difficult to optically monitor and precisely quantify the chromophores concentration alterations using only RGB channels in photography.^21^ In comparison, the CIELab color system was designed based on psychophysical experiments to quantify colors and brightness in a way that is photometrically accurate.^20^ The feature of brown spots is enhanced by the combination of the selected data from the yellow channel of the polarized white light, the luminance channel of the green image, and the blue channel of the blue image in the Lab color space. In addition, it is suggested that higher resolution images (Table 1) and well‐evolved algorithms for image processing and feature identification attributed to the accuracy of CSKIN as well. VISIA is implemented with the RBX (red/brown/X) technology from Canfield, which allows a semiquantitative assessment of specific skin chromophores. However, CSKIN has implemented a modulated algorithm in specific of the feature of the hyperpigmentation, which usually presents as patches rather than punctiform macules in patients with pigmented skin diseases, such as melasma and Nevus of Ota. During the data processing, the color distinction is enhanced and analyzed between the brown area and the normal skin area, and further calculation is added for the parameter optimization.

To set up a reliable standard for comparison, not only the evaluation criteria were unified, but also the evaluation conditions for the patients were also strictly controlled. These factors were considered to have contributed to the fairly high consistency in the visual score graded by dermatologists in the study. However, even on such unified conditions, the internal consistency of the grading results was not satisfying. In clinical practice, the evaluation by a doctor could be subject to a variety of factors apart from unstandardized evaluating standards, such as different illuminations, varied clinical experience, and other subjective factors, and the feedback of the patient.^21^ Therefore, the introduction of imaging techniques does provide a reference value that could guide clinicians in determining individualized therapy or to monitor response.

Both VISIA and CSKIN provide a brief report for the clinician and patients with all types of the captured images and detailed values for corresponding features, which indicated potential to aid patient education. In a survey carried out by Goldsberry et al.,^14^ 86% of the subjects in the investigation reported to have been helped by VISIA to understand their initial concern and to notice their skin problems. There are some factors that contribute to the applicability of CSKIN. The iPad, a portable digital device, works as a control panel by connecting to the photo booth of CSKIN added convenience for operating. Besides, “Cloud” data have made it more available for patient management and statistical analysis. As reported by Chen et al.,^13^ CSKIN also showed stronger capacity in the assessment of erythema compared with VISIA. However, further clinical trials are needed to verify the evaluation ability of other parameters of VISIA and CSKIN, including wrinkles, pores, UV spots, and porphyrins.

## 5 CONCLUSION

Both CSKIN and VISIA showed a fairly good assessing capability in the pigmented areas of the patients. The major parameter in measuring hyperpigmentation by CSKIN presented with a higher correlation with visual grading by the dermatologists in comparison with that of VISIA. Therefore, it is suggested that CSKIN might perform a better role in the facial hyperpigmentation assessment and could serve as a reasonable alternative in the evaluation and follow‐up management of skin disease with hyperpigmentation.

## CONFLICT OF INTEREST

The authors have declared no conflict of interest.

## Data Availability

The data that support the findings of this study are available on request from the corresponding author. The data are not publicly available due to privacy or ethical restrictions.

## References

[1] Yoo J . Differential diagnosis and management of hyperpigmentation. Clin Exp Dermatol. 2022;47(2):251‐258. 33999447 10.1111/ced.14747

[2] Banner A , Dinsey M , Ezzedine K , Dadzie OE . The spectrum of skin diseases occurring in a multiethnic population in north‐west London, U.K.: findings from a cross‐sectional descriptive study. Br J Dermatol. 2017;176(2):523‐525. 27343478 10.1111/bjd.14824

[3] Davis EC , Callender VD . Postinflammatory hyperpigmentation: a review of the epidemiology, clinical features, and treatment options in skin of color. J Clin Aesthet Dermatol. 2010;3(7):20‐31. PMC2921758 20725554

[4] Pawaskar MD , Parikh P , Markowski T , McMichael AJ , Feldman SR , Balkrishnan R . Melasma and its impact on health‐related quality of life in Hispanic women. J Dermatol Treat. 2007;18(1):5‐9. 10.1080/09546630601028778 17365259

[5] Ikino JK , Nunes DH , Silva VPMD , Fröde TS , Sens MM . Melasma and assessment of the quality of life in Brazilian women. An Bras Dermatol. 2015;90(2):196‐200. 25830989 10.1590/abd1806-4841.20152771 PMC4371668

[6] Pandya AG , Hynan LS , Bhore R , Riley FC , Guevara IL , Grimes P . Reliability assessment and validation of the melasma area and severity index (MASI) and a new modified MASI scoring method. J Am Acad Dermatol. 2011;64:78‐83. 20398960 10.1016/j.jaad.2009.10.051

[7] Prendergast PM . Skin Imaging in Aesthetic Medicine. Springer; 2012.

[8] Taylor S , Westerhof W , Im S , Lim J . Noninvasive techniques for the evaluation of skin color. J Am Acad Dermatol. 2006;54(suppl52):S282‐S290. 16631969 10.1016/j.jaad.2005.12.041

[9] Matias AR , Ferreira M , Costa P , Neto P . Skin colour, skin redness and melanin biometric measurements: comparison study between Antera(®) 3D, Mexameter(®) and Colorimeter(®). Skin Res Technol. 2015;21:346‐362. 25645051 10.1111/srt.12199

[10] Clarys P , Alewaeters K , Lambrecht R , Barel AO . Skin color measurements: comparison between three instruments: the Chromameter(R), the DermaSpectrometer(R) and the Mexameter(R). Skin Res Technol. 2000;6(4):230‐238. 11428962 10.1034/j.1600-0846.2000.006004230.x

[11] Wang X , Shu X , Li Z , Huo W , Zou L , Tang Y . Comparison of two kinds of skin imaging analysis software: VISIA(®) from Canfield and IPP(®) from Media Cybernetics. Skin Res Technol. 2018;24:379‐385. 29377397 10.1111/srt.12440

[12] Bossart S , Cazzaniga S , Willenberg T , Ramelet A , Baumgartner M , Hunger RE . Skin hyperpigmentation index: a new practical method for unbiased automated quantification of skin hyperpigmentation. J Eur Acad Dermatol Venereol. 2020;34:e334‐e336. 32103550 10.1111/jdv.16312 PMC7496784

[13] Chen Y , Hua W , Li A , He H , Xie L , Li L . Analysis of facial redness by comparing VISIA(®) from Canfield and CSKIN(®) from Yanyun Technology. Skin Res Technol. 2020;26(5):696‐701. 32196761 10.1111/srt.12856

[14] Goldsberry A , Hanke CW , Hanke KE . VISIA system: a possible tool in the cosmetic practice. J Drugs Dermatol. 2014;13:1312‐1314. 25607694

[15] Pascoe VL , Enamandram M , Corey KC , Cheng CE , Javorsky EJ , Sung SM , Donahue KR , Kimball AB . Using the Physician Global Assessment in a clinical setting to measure and track patient outcomes. JAMA Dermatol. 2015;151(4):375‐81. 25549367 10.1001/jamadermatol.2014.3513

[16] Bossart S , Cazzaniga S , Willenberg T , Ramelet AA , Heidemeyer K , Uthoff H , Baumgartner M , Hunger RE , Seyed Jafari SM . Reliability assessment and validation of the skin hyperpigmentation index compared to the physician global assessment score. Dermatology. 2022;238(4):688‐91. 34969031 10.1159/000520753

[17] Vashi NA , Wirya SA , Inyang M , Kundu RV . Facial hyperpigmentation in skin of color: special considerations and treatment. Am J Clin Dermatol. 2017;18(2):215‐230. 27943085 10.1007/s40257-016-0239-8

[18] Xie W , Pakdel E , Liang Y , Kim YJ , Liu D , Sun L . Natural eumelanin and its derivatives as multifunctional materials for bioinspired applications: a review. Biomacromolecules. 2019;20(12):4312‐4331. 31696698 10.1021/acs.biomac.9b01413

[19] Weatherall IL , Coombs BD . Skin color measurements in terms of CIELAB color space values. J Invest Dermatol. 1992;99(4):468‐473. 1402005 10.1111/1523-1747.ep12616156

[20] Schanda J . Colorimetry: Understanding the CIE System. Wiley Interscience; 2007.

[21] Abdlaty R , Hayward J , Farrell T , Fang Q . Skin erythema and pigmentation: a review of optical assessment techniques. Photodiagn Photodyn. 2021;33:102127. 10.1016/j.pdpdt.2020.102127 33276114

